# Ag/Au/Polypyrrole Core-shell Nanowire Network for Transparent, Stretchable and Flexible Supercapacitor in Wearable Energy Devices

**DOI:** 10.1038/srep41981

**Published:** 2017-02-03

**Authors:** Hyunjin Moon, Habeom Lee, Jinhyeong Kwon, Young Duk Suh, Dong Kwan Kim, Inho Ha, Junyeob Yeo, Sukjoon Hong, Seung Hwan Ko

**Affiliations:** 1 Applied Nano and Thermal Science Lab, Department of Mechanical Engineering, Seoul National University, 1 Gwanak-ro, Gwanak-gu, Seoul, 08826, Korea; 2 Department of Physics, Kyungpook National University, 80 Daehak-ro, Pook-gu, Daegu 41566, Korea; 3 Department of Mechanical Engineering, Hanyang University, 55 Hanyangdaehak-ro, Sangnok-gu, Ansan, Gyeonggi-do, 15588, Republic of Korea

## Abstract

Transparent and stretchable energy storage devices have attracted significant interest due to their potential to be applied to biocompatible and wearable electronics. Supercapacitors that use the reversible faradaic redox reaction of conducting polymer have a higher specific capacitance as compared with electrical double-layer capacitors. Typically, the conducting polymer electrode is fabricated through direct electropolymerization on the current collector. However, no research have been conducted on metal nanowires as current collectors for the direct electropolymerization, even though the metal nanowire network structure has proven to be superior as a transparent, flexible, and stretchable electrode platform because the conducting polymer’s redox potential for polymerization is higher than that of widely studied metal nanowires such as silver and copper. In this study, we demonstrated a highly transparent and stretchable supercapacitor by developing Ag/Au/Polypyrrole core-shell nanowire networks as electrode by coating the surface of Ag NWs with a thin layer of gold, which provide higher redox potential than the electropolymerizable monomer. The Ag/Au/Polypyrrole core-shell nanowire networks demonstrated superior mechanical stability under various mechanical bending and stretching. In addition, proposed supercapacitors showed fine optical transmittance together with fivefold improved areal capacitance compared to pristine Ag/Au core-shell nanowire mesh-based supercapacitors.

Recent progresses in wearable electronics have led to the birth of various flexible and stretchable electronics such as flexible displays and stretchable heaters in their prototype forms^1^,^2^,^3^,^4^. In the meantime, since all electronics require an energy source, development of flexible and stretchable energy storage devices is urgently needed for further advances in wearable electronic devices. Supercapacitors have become a viable candidate for wearable electronics as a complementary energy storage source with the conventional battery due to advantages such as quick charging time (from a few seconds to several minutes), a substantial life cycle, high power density^5^,^6^,^7^,^8^,^9^, lightweight, no risk of explosion, and especially high compatibility over large mechanical deformations that are incorporated in general human motion^10^,^11^,^12^,^13^.

Lately, there have been many attempts to fabricate transparent, flexible, and stretchable (TFS) supercapacitors. Most TFS supercapacitors used nanomaterials such as carbon nanotubes (CNT), graphene, and metal nanowires to enable flexible motion. Recent research trends have been focused on not only the flexibility and stretchability enhancement, but also improving the specific capacitance by making interconnected nano-cup morphologies or changing surface properties using redox-active materials^14^,^15^,^16^,^17^. The conducting polymer, which is typically electropolymerized on the electrically conductive substrate, is considered one of the most promising candidates to improve the specific capacitance of the supercapacitor, owing to its reversible faradic redox nature. Furthermore, its fabrication process does not require binders or additives, consequently facile and efficient ion transport during redox reaction is enabled^18^. Ge *et al*.^19^ and Lin *et al*.^20^ have reported on transparent and flexible supercapacitors with improved specific capacitance through electropolymerization of conducting polymer on the surface of CNT film. However, such electropolymerization processes on the transparent CNT films typically require extra steps, such as employing electropolymerization on a fluorine-doped tin oxide (FTO) substrate and subsequent transfer to flexible substrate or growing aligned CNT to fabricate transparent films with high electrical conductivity. Given that these extra steps for the electropolymerization increase the overall complexity in the fabrication process which is inevitably related to the fabrication time and cost, a more efficient platform is required for the facile fabrication of conducting polymer electrode-based high-performance TFS supercapacitor. Here, as a new platform, we suggest a silver nanowire (Ag NW) percolation network as a base electrode material to improve the transparency and stretchability of the suggested TFS supercapacitors. In addition to excellent electrical conductivity and optical transparency, both flexibility and stretchability have been successfully demonstrated in previous studies using Ag NW as a base electrode material^21^,^22^,^23^. Besides, the fabrication processes for preparing Ag NW network structure such as Meyer rod coating, vacuum filtration and spraying method are fast, cost-effective and scalable with easy application to large-area devices^24^,^25^,^26^. However, no research have been conducted on direct application of Ag NW networks as a current collector of TFS supercapacitors because conducting polymer’s redox potential for electropolymerization is higher than that of Ag which makes the Ag NW chemically incompatible for direct electropolymerization.

In an effort to realize an advanced TFS supercapacitor for wearable electronics application, we developed a direct electropolymerization of conducting polymer method on a metal nanowire network by introducing an ultrathin gold layer sheath on Ag NW to achieve an Ag/Au/conducting polymer core-shell NW. It was confirmed that the electrochemical stability of Ag NW is significantly enhanced after covering its whole surface with a gold layer of 3–5 nm through galvanic replacement-free deposition, while maintaining the electrical conductivity and optical transmittance of its percolation network^27^. Since the standard reduction potential of gold (+1.50 V) is higher than electropolymerizable monomer (e.g., Pyrrole, +0.8 V)^28^, we confirmed in this study that Ag/Au core-shell NW can successfully undergo the electropolymerization process, resulting in an Ag/Au/conducting polymer core-shell NW mesh without damaging the original nanowire. Based on the resulting Ag/Au/conducting polymer NW network films, we assembled a new type of TFS supercapacitor, which shows not only the merits of a metal nanowire network electrode such as high electrical conductivity, optical transparency, flexibility, and stretchability but also greatly enhanced specific capacity compared to the previously reported TFS supercapacitors.

## Results and Discussions

**Figure 1:**
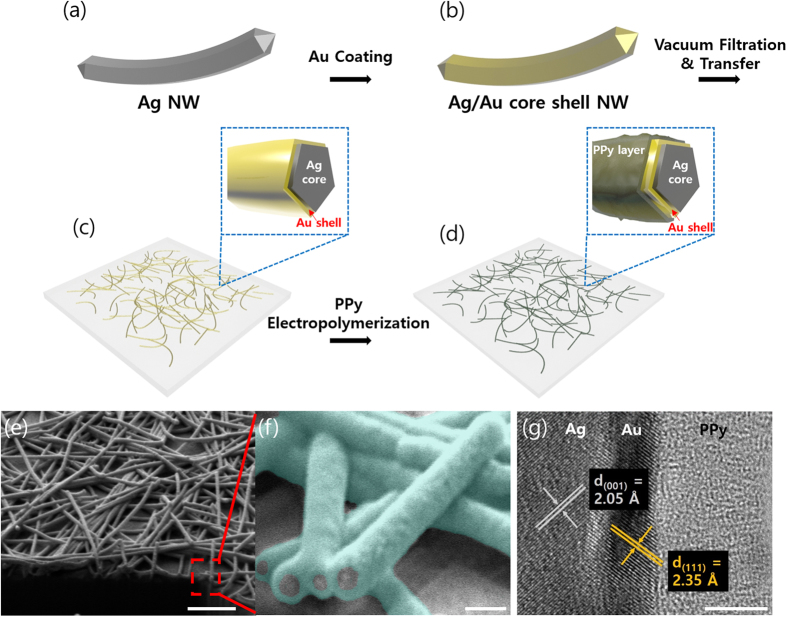
Fabrication step of Ag/Au/PPy core-shell NW network mesh film and its micro and nanoscale structure. (**a**,**b**) Schematics of synthesized pristine Ag NW and Ag/Au core-shell NW. (**c**) Schematic of Ag/Au core-shell NW network mesh film and (**d**) Ag/Au/PPy core-shell NW mesh film produced by electropolymerization of pyrrole. (**e**,**f**) FIB analysis of Ag/Au/PPy core-shell NW on PET substrate. Cyan pseudo color shows PPy coated on the Ag/Au NW surface. (**g**) High resolution TEM image of core-shell structure where each region represents Ag, Au, and PPy. Scale bars in (**e**,**f**,**g**) show 1 μm, 100 nm, and 5 nm respectively.

In this research, we used polypyrrole (PPy) as an active polymer layer due to its superior thermal stability, fast charge-discharge mechanism, and mild electropolymerization condition^29^,^30^,^31^,^32^,^33^. Figure 1 shows the whole fabrication process of Ag/Au/PPy core-shell NW network based transparent conducting film. This process includes three simple steps. First, pristine Ag NWs with a diameter of 30 nm were coated with a thin gold layer. This synthesis process is conducted by injecting a gold precursor slowly with a syringe pump into the Ag NW solution containing a capping agent, reducing agent and pH-increasing agent^27^. Detailed experimental conditions, including injection rate and molar concentration of each chemical, are described in the Experimental Section. Subsequently, Ag/Au core-shell NWs dispersed in the ethanol were transferred onto a polyethylene terephthalate (PET) substrate through the vacuum filtration method, which results in a well-connected nanowire percolation network with high electrical conductivity as well as optical transparency (Fig. 1(c)). Electrical conductivity and optical transparency of the NW based electrode can be easily altered by adjusting the concentration of Ag/Au NW solution in the filtration step. In this work, the density of Ag/Au NW was fixed to 12.5 μg/cm^2^ which guarantees both electrical conductivity and transparency of the NW based electrode. After that, the Ag/Au core-shell NW network electrode was immersed in pyrrole-dissolved solution for electrical coating of PPy through cyclic potential sweep (Fig. 1(d)). All of the electropolymerization process was conducted under the same condition (a scan rate of 0.2 V/sec ranging from 0 to 1.0 V) except for the number of voltage sweep cycles to control the thickness of the PPy sheath layer. Owing to the core Ag NW, the obtained Ag/Au/PPy core-shell NW mesh film still showed high electrical conductivity (10–30 Ω/sq). According to the previous research that used a FTO glass to enable uniform coating of conducting polymer on the surface of transparent CNT network film, direct electropolymerization on a CNT percolation network without a conducting substrate results in non-uniform polymer coating due to significant in-plane voltage drop^19^. On the contrary, the direct electropolymerization on the Ag/Au core-shell NWs over the entire substrate of 4 cm in diameter was possible owing to its low sheet resistance.

**Figure 2:**
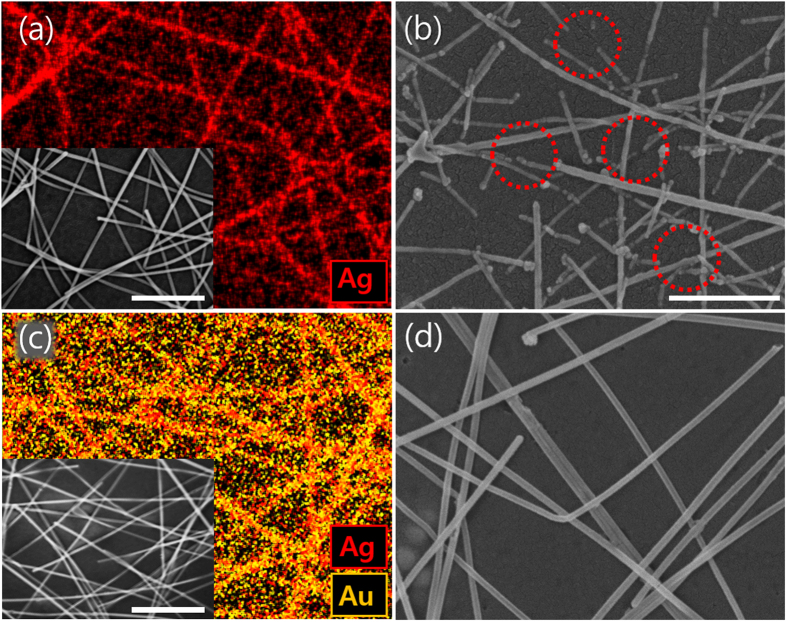
Electrochemical stability test upon potential scan. (**a**,**c**) EDX images of Ag NWs and Ag/Au core-shell NWs on the PET substrates. Insets are SEM images of corresponding samples. (**b**,**d**) SEM images of Ag NWs and Ag/Au core-shell NWs on the PET substrate that experienced cyclic voltage sweep from 0 to 1.0 V. Red dotted circles in (**b**) clearly represent disconnected nanowires. All scale bars show 500 nm.

To confirm the synthesized Ag/Au/PPy core-shell NW network structure, we performed a focused ion beam (FIB) cross sectional imaging analysis on Ag/Au/PPy NW mesh film that underwent 15 cycles of PPy coating. As shown in Fig. 1(e), the PPy was conformally coated on the Ag/Au core-shell NW surfaces and the diameter of the resulting Ag/Au/PPy NW was approximately 100 nm. As shown in Fig. 1(f), FIB analysis revealed the existence of core Ag (grey pseudo-colored) without any observable damage, even after the electropolymerization process, while the PPy polymer shell (cyan pseudo-colored) was coated uniformly over the Ag/Au core-shell NW network. The core-shell structure was more closely examined with a transmission electron microscopy (TEM) analysis. The specimen for TEM analysis was prepared by scraping out the Ag/Au/PPy core-shell NWs on the glass substrate and directly attaching them on the holey carbon grid. Figure 1(g) shows the TEM image of the Ag/Au/PPy core-shell NW structure. The periodic lattice distance of 2.05 Å measured from the image processing corresponded to [001] direction of single crystalline Ag. In the TEM image, the Au shell seems slightly darker than the Ag core part, which implies that there are heavy atoms as compared to the Ag core region. The measured lattice distance also matches to [111] direction of single crystalline Au, verifying the presence of a Au outer shell. The Au atoms conformally covered the surface of Ag NW, and the deposition thickness is approximately 3 nm. Lastly, the Au shell covered with PPy shows an amorphous structure from the TEM image. As a result, it is confirmed that the thin Au outer shell coated on the surface of Ag NW inhibits the oxidation of Ag NW during the electropolymerization process. According to the standard reduction potential, Ag is oxidized at the potential of 0.8 V, and the oxidation of pyrrole and aniline monomer occurs at 0.8 and 1.0 V, respectively^28^,^34^. Therefore, it is impossible to implement electropolymerization of PPy directly on the surface of Ag NWs. However, the redox potential of Au is 0.7 V higher than that of pyrrole monomer and Ag, thus preventing oxidation of Ag NW and enabling polymerization on the surface of Ag/Au core-shell NWs. To show the effectiveness of the thin Au coating in oxidation of core materials, we compared the Ag NW mesh film and Ag/Au core-shell NW mesh film. Figure 2(a and c) show energy-dispersive X-ray spectroscopy (EDX) mappings of Ag NWs and Ag/Au core-shell NWs, respectively. Although their initial morphologies exhibit no apparent differences, as shown in their corresponding inset scanning electron micrograph (SEM) images, the EDX result confirms that the gold elements are found along the NW only in the case of Ag/Au core-shell NW. Figure 2(b and d) show the SEM images of Ag NW and Ag/Au core-shell NW, respectively, after a single cycle of the electropolymerization process. In contrast to Ag NW, which shows disconnection of the nanowire network upon electropolymerization (red dotted circles in Fig. 2(b)), the Ag/Au core-shell NW shows no observable damage, validating the effectiveness of the Au sheath layer in protecting the core Ag NW.

**Figure 3:**
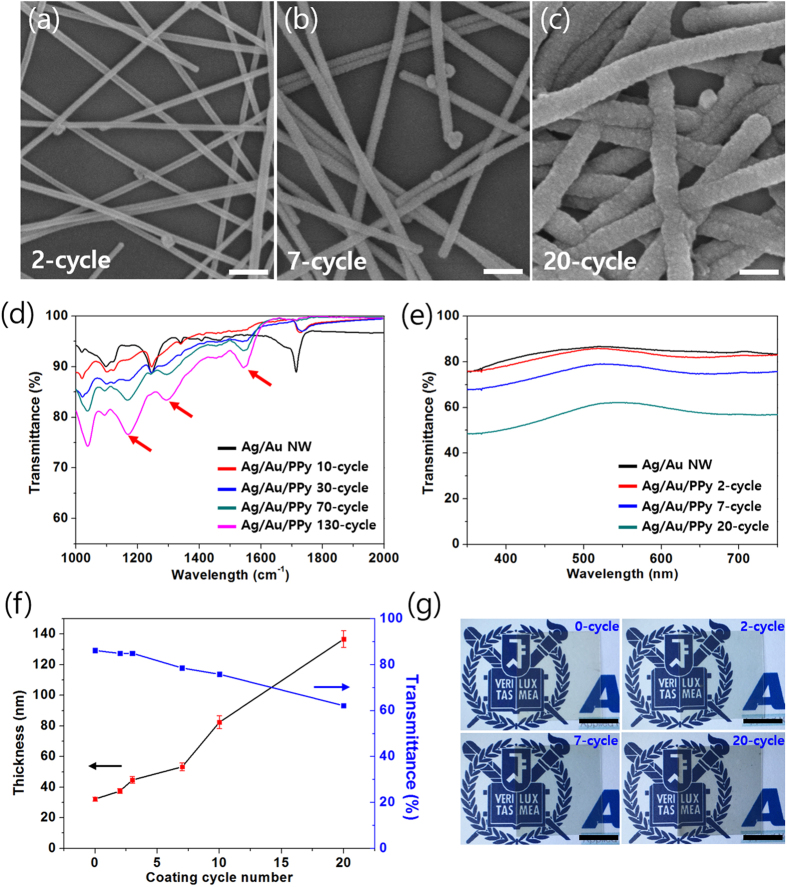
(**a**–**c**) SEM images of Ag/Au/PPy core-shell NWs with increasing amount of PPy coated on the Ag/Au NW surfaces according to the PPy deposition cycles. The scale bars show 200 nm. (**d**) FT-IR and (**e**) UV-VIS spectrum of Ag/Au and Ag/Au/PPy core-shell NW samples with different amount of PPy. (**f**) Variation of NW thickness and transmittance depending on PPy coating cycle number. (**g**) Digital images of Ag/Au and Ag/Au/PPy core-shell NW network mesh films. The scale bars indicate 1 cm.

In order to determine the amount of coating that is appropriate for a transparent supercapacitor, and to obtain the relationship between thickness of the deposited PPy and transmittance, we fabricated Ag/Au/PPy core-shell NW mesh on PET films, while increasing the number of coating cycles from 2 to 20. Figure 3(a–c) show the SEM images of the Ag/Au/PPy core-shell NW corresponding to the coating cycles of 2, 7, and 20. As can be seen in these SEM images, the NW thickness gradually increased, covering the NW mesh surfaces with thicker PPy, and this trend was maintained as the coating cycles increase up to 130 cycles. The SEM images in Figure S1 show Ag/Au/PPy core-shell NW network with the increasing diameter according to the number of cyclic potential sweep. The presence of PPy was further proved by Fourier transform infrared (FT-IR) spectroscopy (Fig. 3(d)), in which specific bands (marked with red arrows) corresponding to polaron and bipolaron absorptions^35^ were enhanced as specimens of Ag/Au/PPy core-shell NW network mesh films with larger diameters were measured.

Figure 3(e) shows the UV-VIS spectrum of corresponding transparent and conducting films. The film that consists of Ag/Au core-shell NWs of 12.5 μg/cm^2^ density showed 86% transmittance at 550 nm. And the transmittance of Ag/Au core-shell NW network mesh subjected to 2 to 20 cyclic scans for electropolymerization decreased from 85% to 62% at 550 nm. The relationship between the coating cycles and thickness of NWs as well as transmittance at 550 nm, is illustrated in Fig. 3(f). As the number of coating cycles increased from 2 to 20, the average diameter increased from 32 to 136 nm, and the transmittance was correspondingly reduced by 24%. The resulting film images are shown in Fig. 3(g), and the darker film can clearly be seen as the amount of coating increases. The weight of the PPy loaded on the Ag/Au core-shell NW electrode can be calculated by monitoring the total charge consumed during the electro-polymerization process. The calculated weight density of 2 cycles, 7 cycles and 20 cycles’ samples are 1.6 μg/cm^2^, 6.3 μg/cm^2^ and 19.8 μg/cm^2^ respectively. These values can be considered as the mass of the active material of the supercapacitor fabricated with the Ag/Au/PPy core-shell NW based electrode. As shown in Figure S2, we fabricated supercapacitor with two identical Ag/Au/PPy core-shell NW electrodes by attaching them with PVA-H_3_PO_4_ gel electrolyte. The supercapacitors fabricated with 2 and 3 coating cycles showed high transmittance of 73% and 64% at 550 nm, and their digital images are shown in Figure S3.

Due to large surface area, the Ag/Au/PPy core-shell NW mesh structure is anticipated to show higher specific capacitance compared to PPy film prepared on the flat substrate. To confirm this, the same amount of PPy was electropolymerized on the surface of Ag/Au core-shell NW mesh film and Au-sputtered quartz substrate. By monitoring the amount of charges used for electropolymerization through the Versa Studio software, the two substrates were coated with the same amount of PPy. As shown in Figure S4(a), although hundreds of nanometer-sized sphere-like PPy aggregates were synthesized on the flat Au-sputtered substrate with rough 2-dimensional surface. On the contrary, the Ag/Au core-shell NW mesh coated with the same amount of PPy shows 3-dimensional NW network structure providing much larger surface area (Figure S4(b)). Figure S4(c) shows the cyclic voltammogram (CV) of the supercapacitor that consists of bare flat Au-sputtered substrates, PPy-coated flat Au substrates and the Ag/Au/PPy core-shell NW mesh film. In conducting polymer based supercapacitors, the charges are stored by the incorporation of anions to the polymer backbone and abstraction of electrons to the current collector^36^. In this experiment, phosphate ions are incorporated with positively charged PPy during the forward CV scan, and they are detached from PPy during the discharging process. Due to this redox process, the full cells made by electrodes of PPy on flat gold substrates (blue line) and Ag/Au/PPy core-shell NW network mesh (red line) presented enhanced CV characteristics at 100 mV/s, however the pristine flat Au substrate showed almost no current density (blue line). More importantly, the enhancement of specific capacitance is clearly shown by approximately three times wider rectangular CV shape of the Ag/Au/PPy core-shell NW based supercapacitor compared to the flat PPy film based supercapacitor.

**Figure 4:**
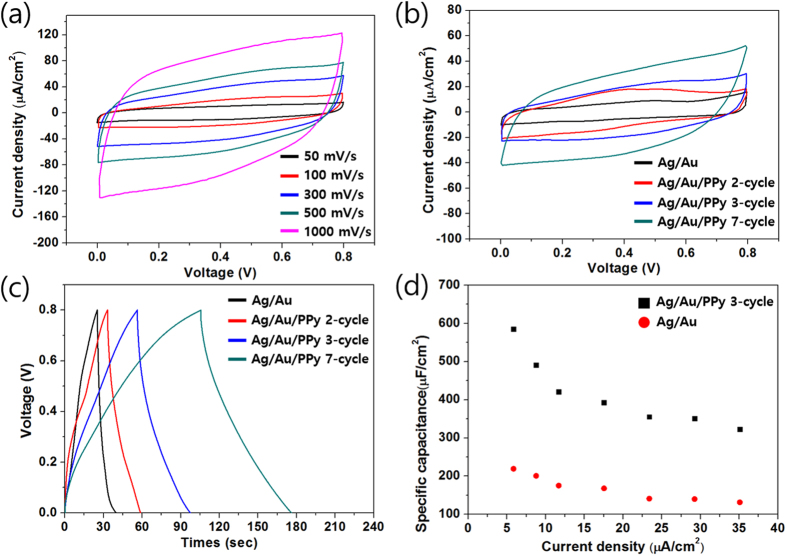
(**a**) CV curves for a supercapacitor based on Ag/Au/PPy (3-cycle) core-shell NW at different scan rates. (**b**) CV curves for supercapacitors with increasing amount of PPy at a scan rate of 100 mV/s. (**c**) Galvanostatic charge/discharge curves for supercapacitors with increasing amount of PPy at a current density of 5.8 μA/cm^2^. (**d**) The areal capacitance of Ag/Au, Ag/Au/PPy (3-cycle) as a function of the current density.

CV test results for Ag/Au/PPy 3-cycle core-shell NW mesh film based supercapacitor at diverse potential scan rates, from 50 mV/s to 1 V/s, are shown in Fig. 4(a). At a scan rate of 50 mV/s, an almost perfect rectangular CV shape was investigated, and this superior CV shape was maintained at a high scan rate of 1 V/s. This rapid current response to the voltage change is attributable to fast electron transport through the core Ag NW mesh and tight coupling between the Au surfaces and PPy as well as conformal PPy coating around the metal nanowires. Furthermore, the fast diffusion of electrolytes through the vacant area in the mesh structure enables a fully rectangular CV shape at a high scan rate. Figure 4(b) compares CVs of the as-fabricated supercapacitors based on Ag/Au and Ag/Au/PPy core-shell NW network mesh with different amounts of PPy coating by varying the number of coating cycles. The CV scans yielded roughly rectangular images in the potential window of 0–0.8 V with higher current density and an enlarged area in proportion to the amount of PPy coating, which implies increased areal capacitance. In addition, the linear profile of the charge-discharge curves in Fig. 4(c) of each as-prepared supercapacitor represent an ideal capacitive performance. Based on the charge/discharge graph and the equation C = 2i/[A(ΔV/Δt)], the areal capacitances were calculated where *A* is the geometric area of the electrode^37^. These values were increased by 75% and 270% as the number of cyclic potential scans for PPy coating increased to 2 and 3 cycles, respectively. This is a huge enhancement in terms of capacitance with minimal transmittance sacrifice of the supercapacitor considering the transmittance changes was from 81% to 77% and 65%. Figure 4(d) shows the areal capacitance of Ag/Au NW mesh and Ag/Au/PPy 3-cycle NW mesh based supercapacitor at various current densities. The areal capacitance decreased from 580 to 320 μF/cm^2^ as current density increased from 5.8 to 35 μA/cm^2^. At such current densities, the Ag/Au/PPy NW supercapacitor maintained 230 to 270% higher areal capacitance than the Ag/Au NW supercapacitor. Figure S5 is the ragone plot which shows energy and power density of the Ag/Au/PPy 3-cycle NW based supercapacitor based on charge-discharge measurement. Both of the energy and power density are comparable to the recently reported transparent^38^ and high-performance^39^ solid-state supercapacitors. Also, the specific capacitance of the supercapacitor considering only the PPy as the active material at the 5.8 μA/cm^2^ current condition was 116.28 F/g. The obtained specific and areal capacitance values are similar or higher than those of many previously reported transparent and flexible supercapacitors using CNT^40^, graphene^15^,^41^,^42^,^43^, and Au mesh with MnO_2_^44^. This is evidently due to the use of conducting polymer that uses the pseudo-capacitance mechanism and 3-dimensional structure of NW network mesh film with enhanced surface area.

**Figure 5:**
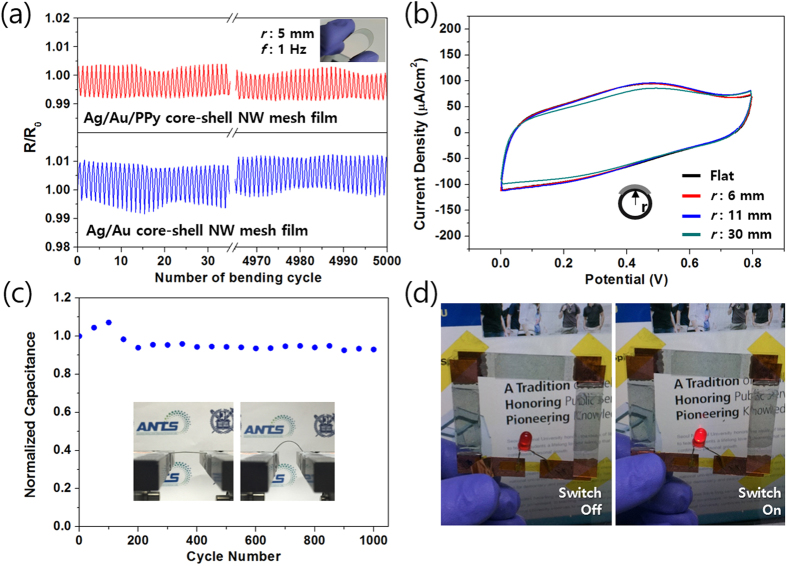
Bending stability test. (**a**) Cyclic bending test comparison of Ag/Au and Ag/Au/PPy core-shell NW film. (**b**) CV curves of the supercapacitor based on Ag/Au/PPy (3-cycle) at a scan rate of 500 mV/s at an indicated bending radii. (**c**) Normalized area-specific capacitance of supercapacitor based on Ag/Au/PPy (3-cycle) as a function of bending cycle number with a radius of 11 mm. Insets are pictures for the flat and bending state of the supercapacitor. (**d**) Demonstration of a series-connected TFS supercapacitor to power a commercial LED.

After confirming the electrochemical properties of fabricated supercapacitors, mechanical reliability under mechanical deformation such as bending and stretching have been investigated. Figure 5 summarizes the reliability of the fabricated supercapacitor based on Ag/Au/PPy core-shell NW mesh film for bending motion and its demonstration for energy devices. First, in order to investigate the stability of NW mesh film upon bending, Ag/Au and Ag/Au/PPy 3-cycle network mesh films were subjected to a repeated bending up to 5,000 cycles. Bending radius and frequency were maintained at 5 mm and 1 Hz during the whole bending test. The relative resistance showed almost no changes between 0.99–1.01 in both cases (red lines for Ag/Au/PPy core-shell NW and blue line for Ag/Au core-shell NW) as shown in Fig. 5(a). This small resistance change under mechanical deformation is attributed to unique NW characteristics, which can easily deform by mechanical bending^45^,^46^. Moreover, this result indicates that PPy coated on the metal surface do not cause any negative effect on tight junction between NWs. Figure 5(b) shows the CV curves of a supercapacitor made by Ag/Au/PPy 3-cycle mesh films under bending conditions ranging from 6 to 30 mm of bending radius. Inferring from the result that the relative resistance remained unchanged, the CV shapes were well retained at any bending radius, signifying that the supercapacitor can be operated without loss of performance. Moreover, the supercapacitor was subjected to cyclic bending (see insets in Fig. 5(c)) and the performance were measured after each 50 bending cycles. For the first 200 bending cycles, the normalized capacitance slightly increased and decreased again, but it retained 93% of its initial capacitance for the next 800 bending cycles (see Fig. 5(c)). Hence, the supercapacitor proposed by the novel Ag/Au/PPy core-shell NW mesh structure is well-suited for flexible electronics that require ultimate stability upon repeatable bending motion. To demonstrate the prepared supercapacitor as a power source for a wearable device, three Ag/Au/PPy 3-cycle NW based supercapacitors were connected in series and mounted on the transparent PET film as shown in Fig. 5(d). The connected supercapacitors lighted a LED after 10 seconds of charging. This confirms the potential of supercapacitors made with Ag/Au/PPy core-shell NW mesh as a wearable device with an improved electrochemical performance as compared to previously reported transparent supercapacitors in terms of mechanical stress from bending cycles.

**Figure 6:**
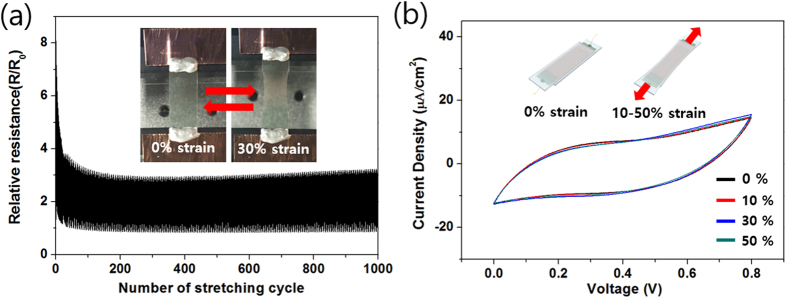
Stretching stability test. (**a**) Strain-dependent relative electrical resistance of Ag/Au/PPy core-shell NW mesh for 1,000 stretching cycles. Insets show digital image of electrical resistance measurement test of Ag/Au/PPy core-shell NW on a PDMS substrate. (**b**) CV curves of the supercapacitor based on Ag/Au/PPy core-shell NW mesh at a scan rate of 50 mV/s at indicated strain rates. Insets are schematic illustration of transparent and stretchable supercapacitor on strain condition.

In addition to bending, the wearable devices require stable performance after repetitive stretching motions^2^. To demonstrate a highly stretchable supercapacitor, Ag/Au/PPy core-shell NW mesh on a stretchable polydimethylsiloxane (PDMS) substrate was fabricated with an identical electrode used in the bending test. As shown in insets of Fig. 6(a), the ends of both PDMS electrodes were connected to copper tape on the horizontally moving stage, and the PDMS substrates were under stepwise cyclic strain from 0 to 30% for 1,000 stretching cycles. Figure 6(a) shows the change of electrical resistance in an Au/Ag/PPy core-shell NW network mesh film. For the first cycle, the relative resistance (R/R_0_) was increased up to 8 at 30% strain, and it recovered to 2 after released from stretched state. As the number of strain cycles was increased to 100, the relative resistance was decreased and eventually reached steady values, which were ~3 and 1 for 30% and 0% strain conditions respectively. The fabricated supercapacitor showed good electrical stability under stretching because the NW network well maintains its original structure^47^. In addition, this result implies that the morphology of PPy on the metal surface was retained as well. Lastly, cyclic potential sweep was conducted to verify the stability of the supercapacitor made with Ag/Au/PPy core-shell NW mesh on PDMS substrate against tensile strain up to 50%, as shown in insets of Fig. 6(b). The CV curves were almost unchanged when the supercapacitor was stretched from 10 to 50% strain. This result demonstrates that the newly proposed Ag/Au/PPy core-shell NW mesh structure can be used as a mechanically stable and stretchable energy device. Moreover, we performed a temperature stability test with the supercapacitor between 20 and 70 °C temperature condition and the results are shown in Figure S6. The supercapacitor exhibited stable performance in the whole temperature region and the capacitance of the supercapacitor slightly increased between 50 and 70 °C, due to the enhancement of the ionic conductivity of the electrolyte^48^,^49^.

## Conclusion

Although Ag NW mesh film has superior electrical conductivity and optical transparency, it has proved difficult to apply its advantages to energy storage devices by forming composite electrodes with conducting polymer, due to the lower redox potential of Ag than that of electropolymerizable monomer. To overcome this problem, we synthesized an Ag/Au core-shell NW through a simple solution process. As the portion of Au is 54 wt% in the core-shell NW, we could reduce the consumption of the high-priced Au up to 46% by using the Ag/Au core-shell NW compared to the ordinary Au NW. Furthermore, due to the electrochemical stability of Au outer shell, electropolymerization of pyrrole monomer could be directly implemented on the Ag/Au core-shell NW network electrode. As a consequence, we successfully made Ag/Au/PPy core-shell NW mesh film and examined the performance as an electrode for transparent and stretchable supercapacitor. Because of PPy that exploits the redox supercapacitor mechanism, this newly proposed supercapacitor showed higher areal capacitance in comparison with previously reported TFS type supercapacitors, such as those based on graphene, CNT and Au network with MnO_2_. In addition, due to the retained merit of Ag NW network structure, the proposed supercapacitor shows stable performance even under repeated bending and stretching circumstance. These research results provide valuable evidence that NW mesh is promising as a transparent, bending, and stretchable electrode for future energy devices.

## Experimental Method

### Preparation of Ag/Au core-shell NWs

The Ag/Au core-shell NWs were prepared through an all-solution process reported previously^27^. Polyvinylpyrrolidone (PVP), L-Ascorbic acid (AA), chloroauric acid (HAuCl_4_), and sodium hydroxide (NaOH) were available from Sigma Aldrich and used without further purification. 1.1 g PVP (M_W_: 55000), 88 mg AA, and 40 mg NaOH were dissolved in 30 mL distilled water, then 100 μL of a 0.5 wt% Ag NW aqueous solution (provided from N&B Co.) was added. In vigorous stirring, 6 mL aqueous solution of HAuCl_4_ (0.15 mM) was slowly injected for 6 hours into the solution. After the injection process, the Ag/Au core-shell NWs were washed by distilled water twice and collected in ethanol.

### Preparation of Ag/Au/PPy core-shell NW mesh film

First, Ag/Au core-shell NW mesh film was prepared by the vacuum filtration method^26^. Simply, Ag/Au core-shell NW solution dispersed in ethanol is poured through a 0.2 μm porous membrane filter. After that, the Ag/Au core-shell NW mesh was transferred to a PET or PDMS substrate by pressing it onto a NW mesh structure on the membrane filter. After that, Ag/Au core-shell NW mesh film was dipped in the 1.0 M KNO_3_ electrolyte solution containing 0.1 M pyrrole for 1 minute. Electrochemical deposition of PPy was made on Ag/Au core-shell NW mesh using platinum counter electrode and saturated calomel electrode (SCE) as a reference electrode. Electropolymerization of pyrrole on the Ag/Au core-shell NW surfaces had been performed through cyclic potential sweep from 0 to 1.0 V at a scan rate of 0.2 V/s. Finally, the composite film was washed with de-ionized water, and dried at room temperature.

### Fabrication of a solid state supercapacitor

Polyvinyl alcohol (PVA) and H_3_PO_4_ were purchased from Sigma Aldrich and used without further purification. Gel electrolyte was prepared by mixing 6 g PVA with 6 g H_3_PO_4_ in 60 g of distilled water at an elevated temperature of 85 °C. Then the Ag/Au/PPy core-shell NW mesh films were immersed in the prepared gel electrolyte for 1 minute and dried for 4 hours in the fume hood. During the drying process, the gel electrolyte was changed to a sticky thin film, and two gel electrolyte-coated electrodes were attached to each other with a moderate pressure to be a solid-state supercapacitor.

### Material characterization and electrical measurement

The morphology, microstructure, and nanostructure of Ag/Au/PPy core-shell NWs were investigated by SEM (JEOL JSM7600), TEM (JEOL JEM-2100F), and FIB (Zeiss AURIGA) measurements. FTIR spectra were obtained using a Nicolet 6700 IR spectrometer. Transmittance and electrical conductivity were measured with a UV-Vis spectrophotometer (Cary 100) and a four-point probe device (M4P 302-System). The cyclic voltammograms and galvanic charge/discharge of the supercapacitor were examined with the two electrodes method through Versa Stat 3. The areal capacitance of the supercapacitors was calculated according to the following equation: C = 2i/[A(ΔV/Δt)], where i, A, ΔV/Δt are the applied current, the slope of the discharge curves after the IR drop, and the area of attached two electrodes.

## Additional Information

**How to cite this article**: Moon, H. *et al*. Ag/Au/Polypyrrole Core-shell Nanowire Network for Transparent, Stretchable and Flexible Supercapacitor in Wearable Energy Devices. *Sci. Rep.* **7**, 41981; doi: 10.1038/srep41981 (2017).

**Publisher's note:** Springer Nature remains neutral with regard to jurisdictional claims in published maps and institutional affiliations.

## Supplementary Material

Supplementary Information
