# Supplementary material for: Ag/Au/Polypyrrole Core-shell Nanowire Network for Transparent, Stretchable and Flexible Supercapacitor in Wearable Energy Devices
Source: Sci Rep. 2017 Feb 3;7:41981. doi: 10.1038/srep41981 (PMC5290463; doi:10.1038/srep41981)
Supplement: Supplementary Information [file srep41981-s1.doc]

Supporting Information

Ag/Au/Polypyrrole Core-shell Nanowire Network

for Transparent, Stretchable and Flexible Supercapacitor

in Wearable Energy Devices

Hyunjin Moon^1†^, Habeom Lee^1†^, Jinhyeong Kwon^1^, Young Duk Suh^1^, DongKwan Kim^1^, Inho Ha^1^, Junyeob Yeo^3^*, Sukjoon Hong^2^*, Seung Hwan Ko^1^*


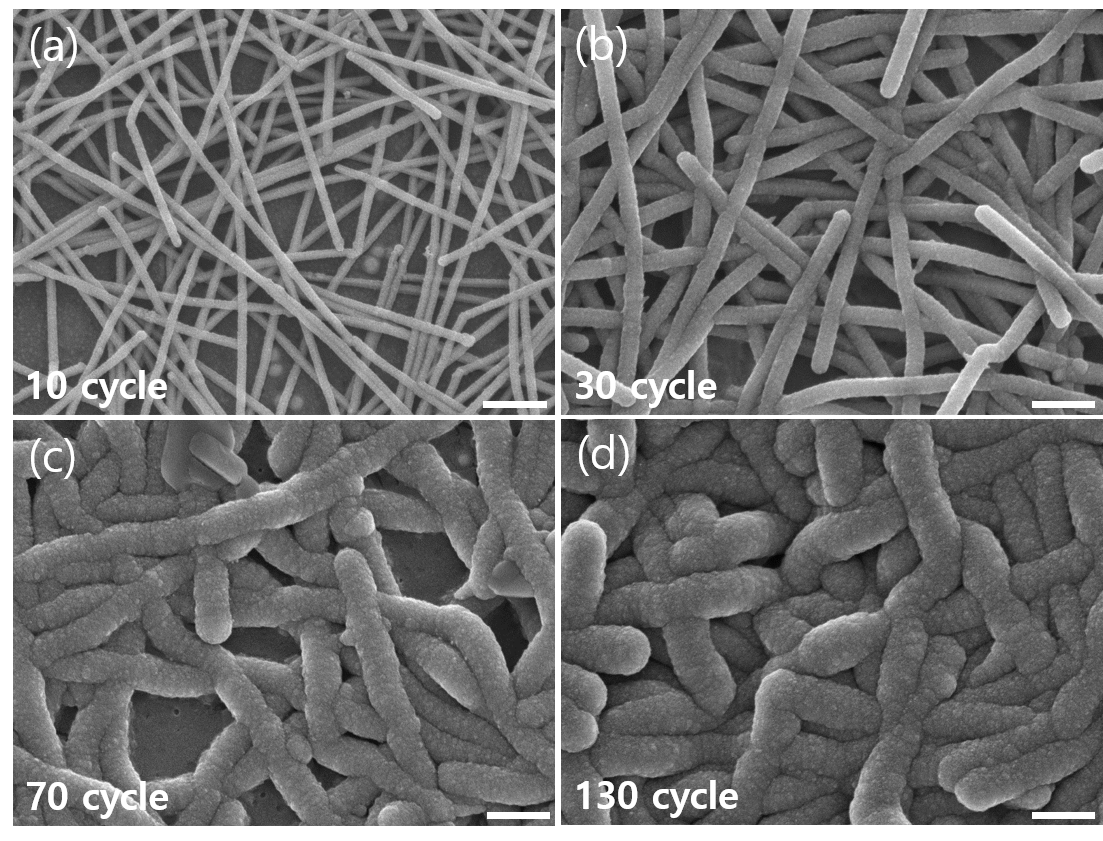


Figure S1. (a-d) SEM images of Ag/Au/PPy core-shell NWs depending on the number of PPy coating cycles. All scale bars represent 500 nm.


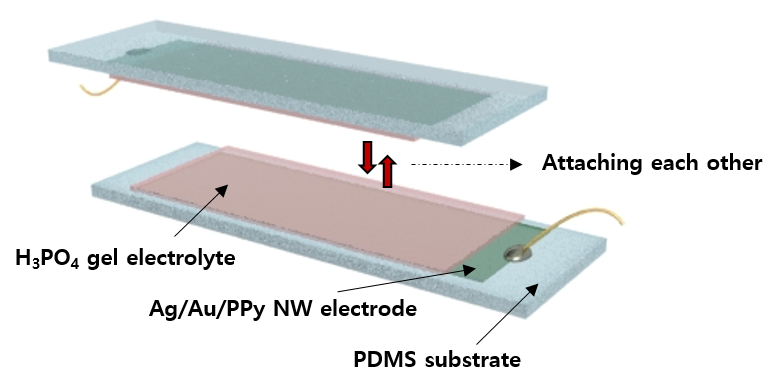


Figure S2. Schematic illustration of Supercapacitor fabrication. The two identical electrodes are attached together by H_3_PO_4_ gel electrolyte film that acts as a glue. Mild pressure is applied on the both sides for another minute. Through this process, a transparent, flexible and stretchable supercapacitor is fabricated.


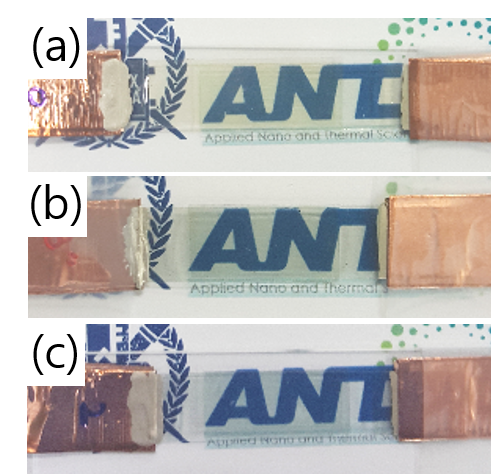


Figure S3. Photograph of a transparent and flexible supercapacitor. (a) Ag/Au core-shell NW-based supercapacitor, (b) Ag/Au/PPy (2-cycle), (c) Ag/Au/PPy (3-cycle) mesh film-based supercapacitors.


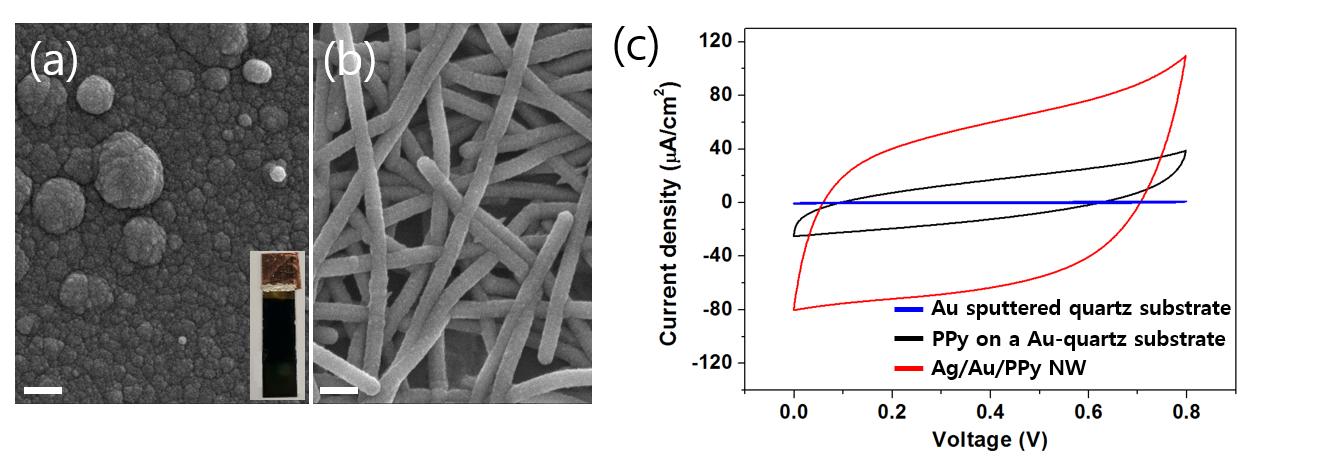


Figure S4. (a) SEM image of PPy coated on the Au-sputtered flat electrode on a quartz substrate. Inset is a digital photograph of PPy-coated Au-sputtered quartz substrate. (b) SEM image of Ag/Au/PPy core-shell NW network. Scale bars in (a) and (b) are 300 nm. (c) Comparison of CV curves among Au-sputtered flat electrode, PPy-coated Au flat electrode and Ag/Au/PPy core-shell NW mesh film.


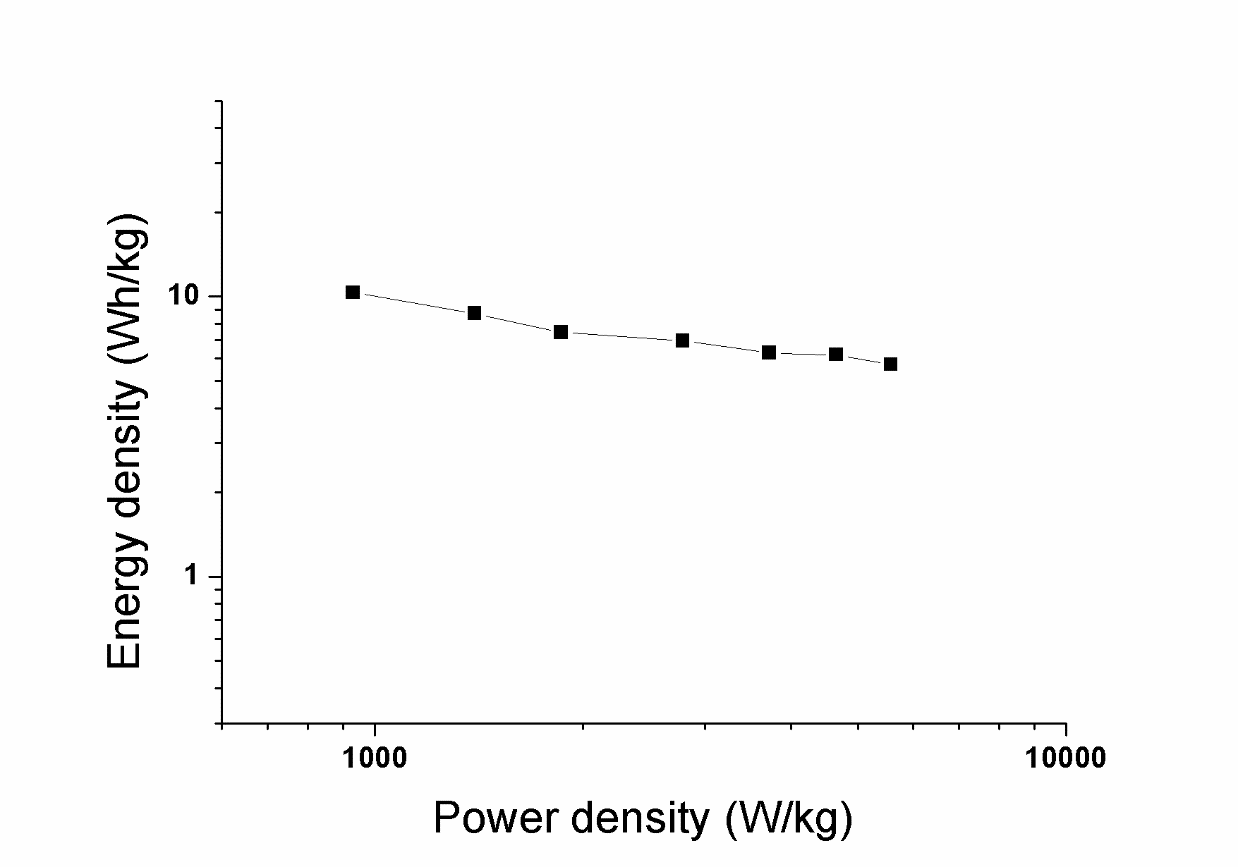


**Figure S5**. Ragone plot of the supercapacitor based on Ag/Au/PPy 3-cycle core-shell NW mesh films.


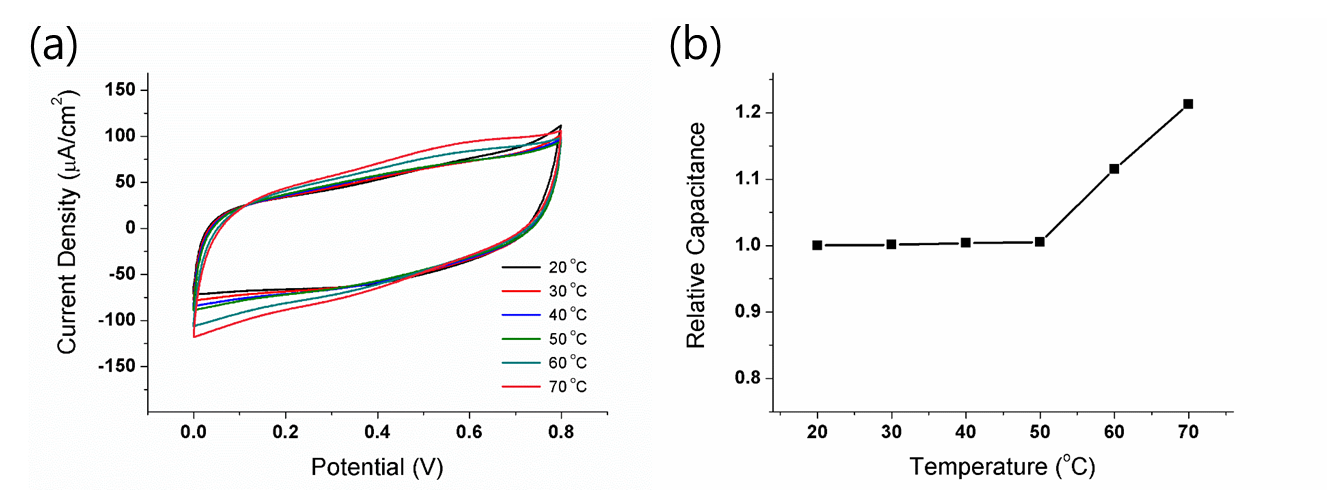


**Figure S6.** **Temperature stability test for the Ag/Au/PPy 3-cycle core-shell NW based supercapacitor.** (a) CV curves at various operating temperature between 20 and 70 °C. (b) Capacitance variation depending on the operation temperature.
